# Transcriptome Profiling Reveals B-Lineage Cells Contribute to the Poor Prognosis and Metastasis of Clear Cell Renal Cell Carcinoma

**DOI:** 10.3389/fonc.2021.731896

**Published:** 2021-08-12

**Authors:** Fafen Yang, Jingjie Zhao, Xiuzhuang Luo, Tong Li, Zechen Wang, Qiuju Wei, Heming Lu, Yiliang Meng, Kai Cai, Liuying Lu, Yushi Lu, Lifen Chen, Suren Rao Sooranna, Linxue Luo, Jian Song, Lingzhang Meng

**Affiliations:** ^1^Center for Systemic Inflammation Research (CSIR), School of Preclinical Medicine, Youjiang Medical University for Nationalities, Baise, China; ^2^Department of Renal Diseases, Affiliated Hospital of Youjiang Medical University for Nationalities, Baise, China; ^3^Life Science and Clinical Research Center, Affiliated Hospital of Youjiang Medical University for Nationalities, Baise, China; ^4^Department of Pediatrics, Affiliated Hospital of Youjiang Medical University for Nationalities, Baise, China; ^5^School of Pharmacy, Youjiang Medical University for Nationalities, Baise, China; ^6^Department of Radiation Oncology, People’s Hospital of Guangxi Zhuang Autonomous Region, Nanning, China; ^7^Department of Oncology, People’s Hospital of Baise, Baise, China; ^8^Radiation Therapy Center , The First Affiliated Hospital of Guangxi University of Chinese Medicine, Baise, China; ^9^Department of Gynaecology and Obstetrics, Affiliated Hospital of Youjiang Medical University for Nationalities, Baise, China; ^10^Department of Metabolism, Digestion and Reproduction, Imperial College London, Chelsea & Westminster Hospital, London, United Kingdom; ^11^Department of Radiation Oncology, Renji Hospital, School of Medicine, Shanghai Jiao Tong University, Shanghai, China

**Keywords:** tumor microenvironment, scRNA-seq, B cells, flow cytometry, metastasis, clear cell renal cell carcinoma

## Abstract

Although immune therapy can improve the treatment of clear cell renal cell carcinoma (ccRCC) significantly, there are still a large proportion of ccRCC patients who progress to metastasis. Targeting the pro-metastatic immune cell in the ccRCC microenvironment could provide a solution to this problem. In this study, B cells in ccRCC biopsies were identified by using scRNA-seq and flow cytometry. The findings indicated the presence of a pro-metastatic B cell type which could be further classified into 3 subpopulations, MARCH3, B2M and DTWD1, based on their large-scaled genetic profiles, rather than traditional Immature/Mature ones. Although all of the 3 subpopulations appeared to contribute to distant metastasis, B cell (B2M) was deemed to be the most essential. Moreover, STX16, CLASRP, ATIC, ACIN1 and SEMA4B, were genes found to be commonly up-regulated in the 3 subpopulations and this was correlated to a poor prognosis of ccRCC. Furthermore, the heterogeneity of plasma cells in ccRCC was also found to contribute to metastasis of the disease. This study offers potential novel therapeutic targets against distant metastasis of cancers, and can help to improve the therapeutic efficiency of ccRCC patients.

## Introduction

Clear cell renal cell carcinoma (ccRCC) is a common tumor of the urinary system which comprises almost 80% of total renal cell carcinomas (1) and usually develops from the nephron epithelial cells (1, 2). Although the clinical treatment of ccRCC has improved significantly with the advent of immunotherapy, metastasis of this type of cancer is still a “bottle neck” that hampers therapeutic efficiency (3). Since it is known that the tumor microenvironment (TME) regulates tumor progression and distant metastasis (4), extensive studies in different cancers including ccRCC (1, 5, 6), have been initiated to explore the intra-tumor cellularity and its related molecules (7, 8), in order to optimize and improve the therapeutic strategies against metastasis.

In recent years, great progress has been made in delineating the heterogeneity of immune cells in ccRCC, especially with respect to the pathological roles played by intra-tumor immune cells. Mapping the TME has shown that numerous immune cell types exist in ccRCC biopsies, including T cells, NK cells, DC, monocytes/macrophages and B cells (6). Further research has also shown that within ccRCC there are exhausted CD8+ T cells which can resist therapeutic intervention, NK cells that exhibit a significant inhibitory immune phenotype due to up-regulated expression of CD48, CD85J, CD45 and PD-1 factors, highly 6-sulfo LacNAc expressing DCs that can predict poor prognosis as well as monocytes/macrophages which exhibit extremely heterogeneous immune phenotypes and genotypes. Previously these would have simply been classified as either pro- or anti- inflammatory cells (6, 9, 10). As for the B cells usually found in ccRCC, due to their low numbers (6), their biological roles in the development of the disease, have long been overlooked.

Recently, it was observed that infiltrated B cells correlate to poor prognosis in renal cancer patients (11), indicating that these cells are implicated in the development of kidney tumors. Therefore, this study was initiated in order to explore the pathological roles of B-lineage cells in ccRCC. Due to their genetic profiles, B cells were classified into 3 subpopulations and plasma cells were classified into 2. The results were validated by flow cytometry analysis of freshly isolated ccRCC biopsies. Furthermore, this study investigated whether the B-linage cells could contributed significantly to ccRCC metastasis.

## Material and Methods

### scRNA-Seq Bioinformatics Analysis

The scRNA-seq data were retrieved from the NCBI GEO database. Expression data of genes relating to healthy kidneys and those of immune cells from ccRCC biopsies were downloaded under the accession IDs GSE131685 (12) and GSE121636 (6), respectively. SCTransform wrapper was used to minimize the technical variations between different panels and platforms. R package Seurat (v4.0.2) was used to analyze scRNA-seq data and a resolution of 0.6 was set for clustering genes/cells. Violin plots and gene nebula maps were used to show specific gene expression levels. Enhanced volcano plots were used to demonstrate differentially expressed genes (DEGs, ccRCC *vs.* control). The gene concept network was analyzed by using R package clusterProfiler (v4.0.0).

### Human Biopsies

To validate the subpopulations of either B or plasma cells, human renal biopsies were dissected after surgery. Control kidney biopsies were collected from patients who underwent radical nephrectomy, and had no clinical record of renal diseases. The human tumor biopsies were isolated from ccRCC patients after surgery.

### Single Cell Preparation

Single cell suspensions were prepared for flow cytometry analysis as described below. After surgery, the biopsies were cut into small pieces in cold PBS and digested with collagenase IV (25mg/mL, Gibco, #17104-019) at 37°C for 40 min. Then PBS/0.5%BSA/2mM EDTA was added and incubated another 10 min after which cells were collected by filtering through a 70-μm stainless strainer.

### Flow Cytometry Analysis

Antibodies used for flow cytometry analysis were: anti-human/mouse CD45 (Invitrogen, # 17-0451-82), anti-human CD20 (Invitrogen, #25-0209-42), anti-human MARCH3 (Invitrogen, #PA5-60351), biotin-anti-B2M (Abcam, #ab269365), anti-human DTWD1 (Invitrogen, #MA5-27492), anti-human IgG1 (Abcam, #ab99776), anti-human RPS12 (Abcam, #ab175219) and anti-human IgG4 (Abcam, #ab1930).

The detailed staining procedure of flow cytometry analysis has been described previously (13). Briefly, nonspecific binding was blocked with Fcγ receptor blocker (Invitrogen, #14-9161-73). For surface staining, cells were incubated on ice with fluorescent-coupled antibodies for 15 min and then washed twice with cold PBS. For intracellular staining, cells were treated using a Cytofix/Cytoperm kit (BD, #554717) according to manufacturer’s introductions and then incubated at 4°C with the relevant fluorescent-coupled antibodies for 15 min. After two washes with cold PBS, cells were re-suspended with PBS/0.5%BSA/2mM EDTA. Samples were then measured with a flow cytometer (Thermal Fisher Attune NxT).

### Statistical Analysis

The results were statistically analyzed using the nonparametric test with Graphpad Prism. P <0.05 was considered statistically significant and *, ** and *** represent *p <*0.05, *p <*0.01 and *p <*0.001, respectively.

## Results

### Identification of B-Lineage Cells

**Figure 1:**
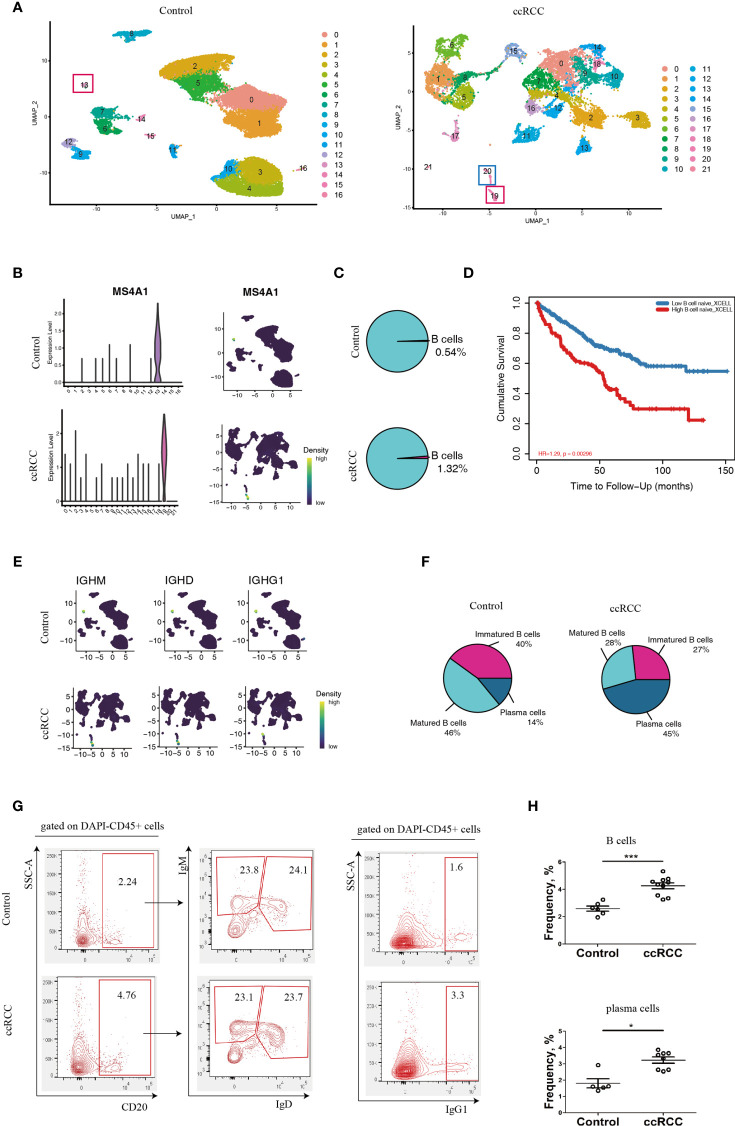
Characterization of B-lineage cells. **(A)** UMAP plots showing the cellular components in control kidneys and ccRCC biopsies. **(B)** Left panel: Violin Plots showing the expression level of MS4A1 in control kidneys and ccRCC biopsies; right panel: gene nebula maps showing the expression pattern of MS4A1 in control kidney and ccRCC biopsies. **(C)** Pie Plots showing the frequency of B cells in control kidneys and ccRCC biopsies. **(D)** Survival curves showing the correlation between B cell density to prognosis. **(E)** Gene nebula maps showing the distribution of B cell development marker genes (IGHM, IGHD and IGHG1) in control kidneys and ccRCC biopsies. **(F)** Pie plots showing the frequency of immature B cells, matured B cells and plasma cells in control kidneys and ccRCC biopsies. **(G)** Flow cytometry analysis of B-lineage cells in control kidneys and ccRCC biopsies. The left panel shows the gating strategy of immature B and matured B cells; the right panel shows the gating strategy of plasma cells. **(H)** Scatter plots showing the statistical analysis of B cells and plasma cells in control kidneys and ccRCC biopsies. Each dot represents one readout. The data represent 3 independent experiments. *p<0.05, ***p<0.001.

With the retrieved scRNA-seq data, the genes from normal human kidney cells and the immune cells from the ccRCC biopsies were grouped into 17 and 22 clusters, respectively (**Figure 1A** and **Supplementary Video 1**). MS4A1, which codes for CD20, was used to identify B cells (14). This gene was preferentially expressed in Cluster 13 in control cells, while it was preferentially expressed in Cluster 19 in ccRCC biopsies (**Figure 1B**), indicating that both these two clusters were B cells. When comparing the frequency of B cells in the control group, it was found to have increased by 2.4 fold in the ccRCC biopsies (**Figure 1C**), indicating that B cells play an important role in the development of cancer. Indeed, a follow-up study showed that the patients with high B cell numbers had a bad survival rate when analyzed from the database using TIMER2.0 (**Figure 1D**). Moreover, immature B cells, which expressed the IGHM gene but not the IGHD as well as matured B cells (expressing IGHD) and plasma cells (expressing IGHG1) (15) were also identified and calculated (**Figures 1E, F**).

Interestingly, the frequency of plasma cells was increased by more than 3.2 fold in ccRCC biopsies when compared to controls, indicating that these cells could potentially play an “active” role in ccRCC. The flow cytometry analysis also showed increases in B and plasma cell frequencies when compared to normal cells (**Figures 1G, H**). This prompted us to further explore the molecules that are expressed by B-lineage cells in ccRCC. However, the frequency of immature and mature B cells was similar in both controls and ccRCC biopsies (**Figures 1F, G**). This led us to seek other methods to explore B cell heterogeneity, rather than to simply classify B cells into either immature or matured phenotypes.

### B Cell Heterogeneity Based on scRNA-Seq Data

**Figure 2:**
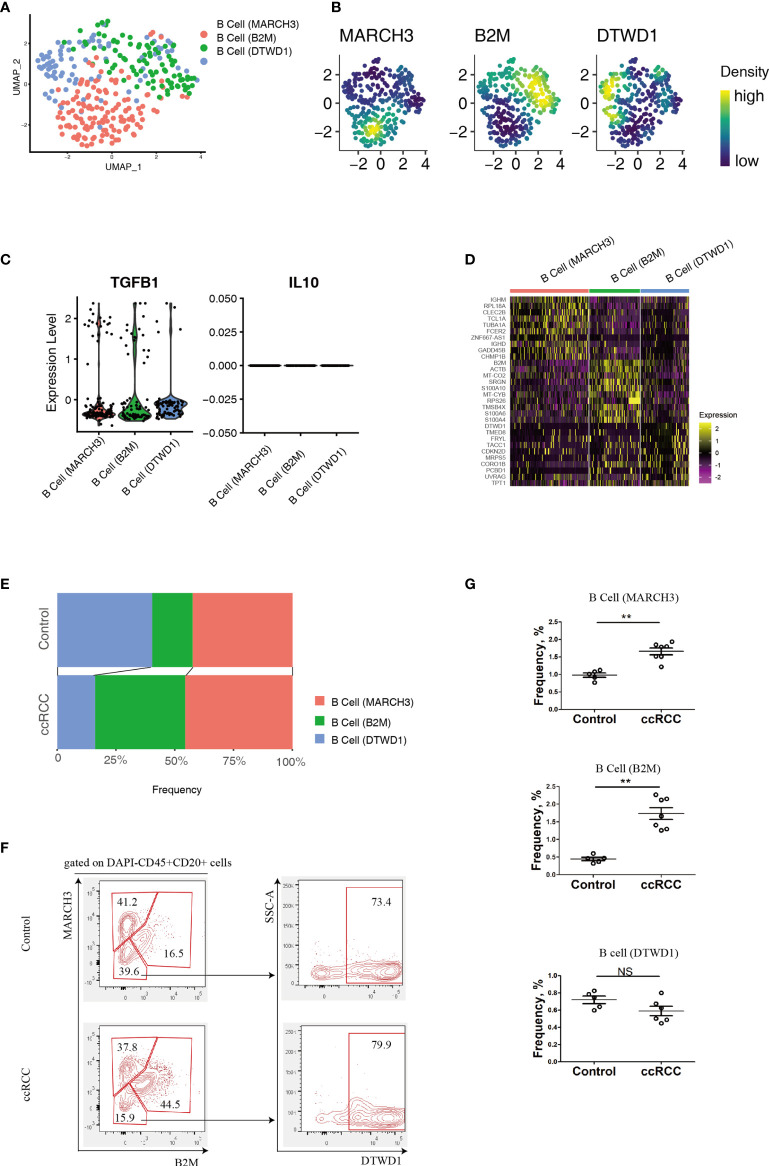
Analysis of B cell Subpopulations By using scRNA-seq And Flow Cytometry. **(A)** A UMAP plot showing the subpopulations of B cells after performing integration of control kidney and ccRCC B cells. **(B)** Gene nebula maps showing the distribution of the three featured genes (MARCH3, B2M and DTWD1) in integrated B cells. **(C)** Violin plots showing the expression of TGF-β1 and IL-10 in B cell subpopulations. **(D)** A heatmap showing the heterogeneity of the top 10 genes expressed among B cell subpopulations. **(E)** Stacked bar plots showing the comparison of B cell subpopulations in control kidneys and ccRCC biopsies. **(F)** Flow cytometry analysis of B cell subpopulations in control kidneys and ccRCC biopsies. **(G)** Scatter plots showing the statistical analysis of B cell subpopulations in control kidneys and ccRCC biopsies. Each dot represents one readout. The data represent 2 independent experiments. **p < 0.01; NS, not significant.

B cell heterogeneity was determine based on their genetic profiles using the scRNA-seq data. After integration of B cells isolated from control and ccRCC biopsies, 3 subpopulation of B cells were found (**Figure 2A**). From the gene nebula maps, 3 genes (MARCH3, B2M and DTWD1) were enriched in specific subpopulations respectively, and each could serve as a feature gene to distinguish the three subpopulations (**Figures 2A, B**). Obviously, these 3 subpopulations produce significant level of TGF-β1, and could produce IL-10 on a baseline level (**Figure 2C**), but neither IL-6 nor IL-17 was detected, indicating these cells could be anti-inflammatory. To explore the disparity of genetic profiles among these 3 subpopulations, the top 10 genes in each subpopulation were exhibited in a heatmap (**Figure 2D**). A significant expression of IGHD in B cell (MARCH) indicated this subpopulation has greater similarity to matured B cells, while the other two subpopulations, B cell (B2M) and B cell (DTWD1), could be sub-classed as immature B cells. Although the heatmap showed the differences of the genetic profiles among these 3 subpopulations, it also indicated that they all shared partial genetic profiles, but with different expression levels of various genes. With genetic profiles, these B cell subpopulations exhibit distinguished biological features, for example, B cell (MARCH3) plays an important role in inducing apoptosis, B cell (B2M) is critical for antigen presentation, and B cell (DTWD1) could potentially modulate the formation of neutrophil extracellular traps (**Supplementary Figure 1A**).

By using a mathematical method, the frequency of the 3 subpopulations were isolated and compared. Stacked bar plots showed that the frequency of the B cell (B2M) increased markedly, indicating this subpopulation is correlated with a poor prognosis and metastasis. However, in these patients, the B cell (MARCH3) subpopulation was increased only slightly and B cell (DTWD1) was decreased (**Figure 2E**). The flow cytometry analysis confirmed that B cells could be separated into 3 subpopulations with the above 3 genes (**Figure 2F**). Furthermore, there was a tendency for the 3 subpopulations of cells to show the same patterns as observed from analysis of the scRNA-seq data (**Figure 2G**).

### B Cell Derived Factors Contributed to Poor Prognosis and Distant Metastasis

**Figure 3:**
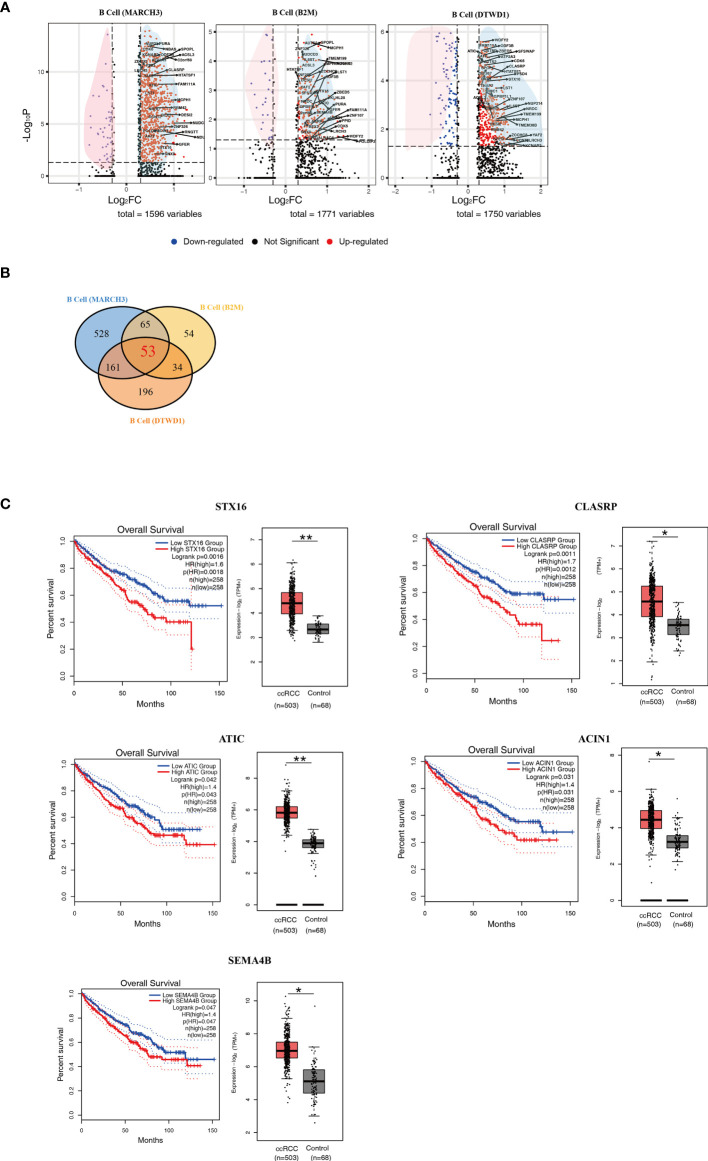
Isolation of Key Genes Associated With Poor Prognosis. **(A)** Volcano plots showing the distribution of common DEGs (ccRCC *vs.* control) among B cell subpopulations. **(B)** A Venn diagram showing the 53 genes up-regulated among the three B cell subpopulations. **(C)** Survival curves showing the 5 up-regulated genes which correlate with poor prognosis in ccRCC patients. In each panel, the expression level of related genes was presented with box plot. *p<0.05; **p<0.01.

In comparison to control kidney cells, newly identified B cell subpopulations showed a large number of differentially expressed genes (DEGs) (**Supplementary Tables 1**–**3**). In this study, those DEGs with *p* < 0.05 and |Log2FC| > 0.3 were considered as significant ones. Volcano plots were adopted to present the DEGs according to the B cell subpopulations (**Figure 3A**). In the plots, the significant DEGs were labelled in either red or blue. In brief, 1596, 1771 and 1750 DEGs were found in the MARCH3, B2M and DTWD1 B cell subpopulations, respectively (**Figure 3A**).

In order to explore those genes relevant to distant metastasis, the lists of DEGs were formatted for gene ontology analysis (**Supplementary Figures 1B–D**). The genetic profiles showed that B cell (MARCH3) in the ccRCC biopsies could potentially contribute to the development of adult fibrosarcoma (**Supplementary Figure 1B**). B cell (B2M) could potentially contributed to adult classical Hodgkin lymphoma, cutaneous T cell lymphoma, Ki1+ anaplastic large cell lymphoma and T cell leukemia (**Supplementary Figure 1C**). It was also found that B cell (DTWD1) could contributed to mantle cell lymphoma (**Supplementary Figure 1D**). The data also showed that B cell (B2M) had more genes that were associated with distant metastasis in ccRCC patients.

In order to find the genes which could contribute to poor prognosis of ccRCC patients, the more commonly expressed genes among the 3 subpopulations were examined and 53 of these were found (**Figure 3B** and **Supplementary Table 4**). These genes were tested in the database Gene Expression Profiling Interactive Analysis (GEPIA), based on long term follow-up studies of ccRCC patients. 5 genes, STX16, CLASRP, ATIC, ACIN1 and SEMA4B, were identified to be the most relevant to poor prognosis of ccRCC patients (**Figure 3C**).

### Plasma Cell Heterogeneity of ccRCC Patients

**Figure 4:**
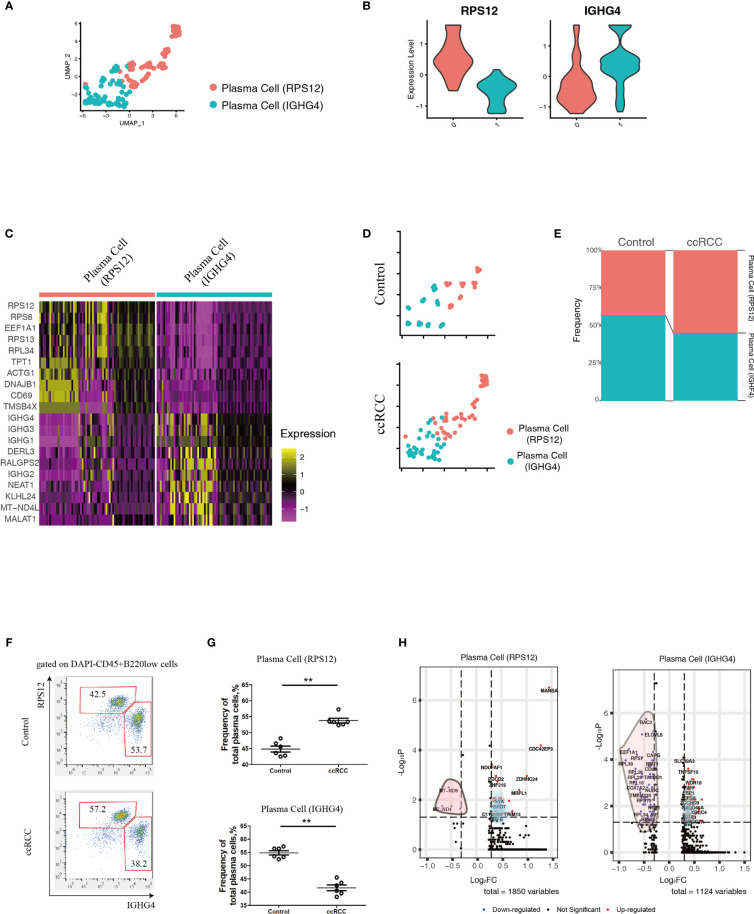
Analysis of Plasma Cell Subpopulations By scRNA-seq And Flow Cytometry. **(A)** A UMAP plot showing the subpopulations of plasma cells after performing integration of control kidney and ccRCC plasma cells. **(B)** Violin plots showing the distribution of the two featured genes (RPS12 and IGHG4) in integrated plasma cells. **(C)** Heatmap showing the heterogeneity of the top 10 genes expressed in the two plasma cell subpopulations. **(D)** Split UMAP plots showing the comparison of plasma cell subpopulations between control kidneys and ccRCC biopsies. **(E)** Stacked bar plots showing the comparison of plasma cell subpopulations between control kidneys and ccRCC biopsies. **(F)** Flow cytometry analysis of plasma cell subpopulations in control kidneys and ccRCC biopsies. **(G)** Scatter plots showing the statistical analysis of the two plasma cell subpopulations in control kidneys and ccRCC biopsies. Each dot represents one readout. The data represent 3 independent experiments. **(H)** Volcano plots showing the distribution of DEGs (ccRCC *vs.* Control) in the two plasma cell subpopulations. **p<0.01.

Using scRNA-seq data, the heterogeneity of plasma cells that contributed to metastasis was also studied. Plasma cells from control kidneys and ccRCC biopsies were integrated and could be sub-clustered into 2 populations (**Figure 4A**) featuring RPS12 and IGHG4 genes, respectively (**Figure 4B**). These plasma cells showed low or no expression of MKI67 (which codes for Ki67, **Supplementary Figure 2**) thus indicating that they were not plasmablasts. A heatmap of the top 10 enriched genes from the two subpopulations showed distinct disparity between their genetic profiles (**Figure 4C**). In comparison to controls, although the frequency of the subpopulations in ccRCC changed slightly, their ratios were reversed. In control cells, the ratio of plasma cell (RPS12)/plasma cell (IGHG4) was less than 1, but in ccRCC biopsies, the ratio was found to be greater than 1 (**Figures 4D, E**). Interestingly, these two subpopulations could also be identified through flow cytometry analysis, and the changes seen were consistent with the scRNA-seq data (**Figures 4F, G**).

In comparison to control kidneys, RPS12 and IGHG4 plasma cells exhibited 1850 and 1124 DEGs, respectively (**Figure 4H** and **Supplementary Tables 5**, **6**). After gene ontology analysis, it was found that plasma cell (RPS12) could potentially contribute to the development of anaplastic carcinoma (**Supplementary Figure 3A**), while plasma cell (IGHG4) could contribute to Burkitt lymphoma (**Supplementary Figure 3B**).

## Discussion

Poor prognosis and metastasis are serious problems in tumor therapy which can often develop from unstable genetics but could also be promoted by the immune environment (16, 17). The advent of immune therapy against tumors, for example, the targeting of PD-1 and CTLA4 signaling pathways (18), have revolutionized clinical treatment of various cancers, including ccRCC (3). However, there are still a significant number of patients who fail to respond to the combined therapy due mainly to malignant metastasis (19). This situation reflects on the urgent need to identify potential new biomarkers for metastasis.

Currently, with respect to metastasis inhibition, most of the promising research have focused on tumor-associated macrophages (TAMs) and has been aimed at targeting on pro-metastatic factors such as CSF1 and CCL2 (16, 20, 21). The basis for this is that the TAMs, could potentially migrate to and participate in the establishment of pre-metastatic niches in distant tissues (22, 23). Although promising results has been obtained (22, 23), there is still a need to develop novel strategies against the pro-metastatic immune cells in the primary tumors.

In this study, the B cells in ccRCC biopsies were isolated and analyzed and it was found that a high density of these cells correlates with poor prognosis of ccRCC patients. This is consistent to a previous report (11). However, up to now, B cells were simply classified as immature/matured cells which referred to their developmental stage. This type of classification was based on the presence of surface markers, such as IgM and IgD. This was mainly due to the limitations of flow cytometry, which could only detect a limited number of possible markers. With new technology, scRNA-seq, has provided the possibility of measuring thousands of markers in a single cell. With the advantage of this technique, in this study, B cells were re-classified into 3 subpopulations based on the similarity/disparity of their genetic profiles (**Figure 2A**), which is a function-related attribute. In addition, these newly reported B cell subpopulations in ccRCC could be identified by flow cytometry with antibodies to specific featured genes. This technique could provide a convenient method for further in-depth research into specific cell lineages in different cancers.

Based on the above method, 5 genes, including STX16, CLASRP, ATIC, ACIN1 and SEMA4B, were identified to be relevant to poor prognosis of ccRCC patients. It has been reported that high serum levels of STX16 was associated with esophageal squamous cell carcinoma (24), CLASRP was associated with head and neck cancers (25), ATIC promoted the development of hepatocellular carcinoma (26) and hyper-methylated ACIN1 was observed in lung adenocarcinoma and acute lymphoblastic leukemia (27, 28). This correlates with the observations from long-term follow-up studies that cancer patients carrying these up-regulated genes exhibited poor prognosis (**Figure 3C**). However, SEMA4B, has been reported to inhibit the growth of some types of non-small lung cancer cells *in vitro* and *in vivo* (29). This contradicts our analysis that patients with low levels of SEMA4B exhibited a better prognosis (**Figure 3C**). It is possible that SEMA4B could play a dual role in the development of certain cancers. Further research should be initiated to delineate the biological roles of SEMA4B. As for the role of plasma cells in ccRCC, this study was able to find 2 subpopulations of these based on their genetic profiles. Although plasma cells comprise only a small percentage of the cells in ccRCC biopsies, it possible to use the ratio of the two types seen as a way to determine the likelihood of forming distant metastases in ccRCC patients.

This study emphasizes the role that B-lineage cells play in primary tumors, and sheds light on discovering key factors relevant to poor prognosis and distant metastasis, which could help identify novel therapeutic target against ccRCC.

## Data Availability Statement

The original contributions presented in the study are included in the article/**Supplementary Material**. Further inquiries can be directed to the corresponding authors.

## Ethics Statement

The studies involving human participants were reviewed and approved by Youjiang Medical University for Nationalities. The patients/participants provided their written informed consent to participate in this study.

## Author Contributions

FY, JZ, and XL collected the biopsies after surgery, and performed flow cytometry analysis. TL and SRS provided the solution for 3D-TSNE plots and partial supplementary figures. ZW, QW, KC, HL, and YM performed partial flow cytometry analysis. LYL, YL, and LC collected the biopsies and performed partial statistical analysis. LM, LL, and JS designed this study, performed scRNA-seq analysis and prepared this manuscript. All authors contributed to the article and approved the submitted version.

## Funding

This research was funded by grants from Guangxi Natural Science Foundation (#2020GXNSFAA259050, #2020GXNSFAA259081 and # 2020GXNSFAA297141), National Science Foundation of China (#31970745), Youjiang Medical University for Nationalities (#yy2019bsky001), High-Level Talent Research Projects of the Affiliated Hospital of Youjiang Medical University for Nationalities (#R20196323 and #R20196346), Wu Jieping Medical Foundation (#2013-428-2081), Research and Development of Appropriate Medical Technology in Guangxi (#S2017082), and the Key Research and Development Program of Gaungxi (#GUIKE-AB17195005).

## Conflict of Interest

The authors declare that the research was conducted in the absence of any commercial or financial relationships that could be construed as a potential conflicts of interest.

## Publisher’s Note

All claims expressed in this article are solely those of the authors and do not necessarily represent those of their affiliated organizations, or those of the publisher, the editors and the reviewers. Any product that may be evaluated in this article, or claim that may be made by its manufacturer, is not guaranteed or endorsed by the publisher.

## Supplementary Material

Supplementary Video 1 3D-TSNE plots showing the cellular components. **(A)** 3D-TSNE plot showing cellular components of control biopsies. **(B)** 3D-TSNE plot showing cellular components of ccRCC biopsies.

Supplementary Figure 1 Gene-Set-Enrichment Analysis of B Cell Subpopulations. **(A)** Dot plot showing the biological features of three B cell subpopulations. **(B)** Analysis of gene-concept-network in B cell subpopulation: B cell (MARCH2). **(C)** Analysis of gene-concept-network in B cell subpopulation: B cell (B2M). **(D)** Analysis of gene-concept-network in B cell subpopulation: B cell (DTWD1).

Supplementary Figure 2 Confirmative Analysis Of Plasma Cells. A violin plot showing the expression levels of MKI67 between plasma cell subpopulations.

Supplementary Figure 3 Gene-Set-Enrichment Analysis of Plasma Cell Subpopulations. **(A)** Analysis of gene-concept-network in plasma cell subpopulation: plasma cell (RPS12). **(B)** Analysis of gene-concept-network in plasma cell subpopulation: plasma cell (IGHG4).
