# Supplementary figures and images for: Transcriptome Profiling Reveals B-Lineage Cells Contribute to the Poor Prognosis and Metastasis of Clear Cell Renal Cell Carcinoma
Source: Front Oncol. 2021 Aug 12;11:731896. doi: 10.3389/fonc.2021.731896 (PMC8416254; doi:10.3389/fonc.2021.731896)

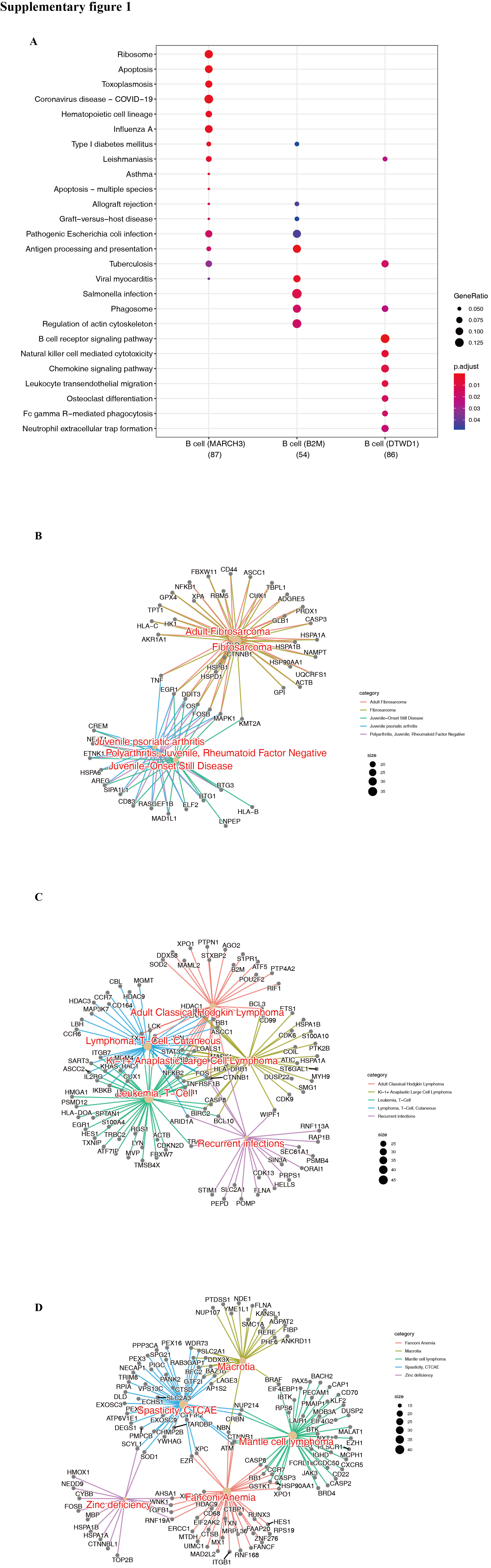

Supplement: Supplementary Figure 1 — Gene-Set-Enrichment Analysis of B Cell Subpopulations. (A) Dot plot showing the biological features of three B cell subpopulations. (B) Analysis of gene-concept-network in B cell subpopulation: B cell (MARCH2). (C) Analysis of gene-concept-network in B cell subpopulation: B cell (B2M). (D) Analysis of gene-concept-network in B cell subpopulation: B cell (DTWD1). [file Image_1.jpg]

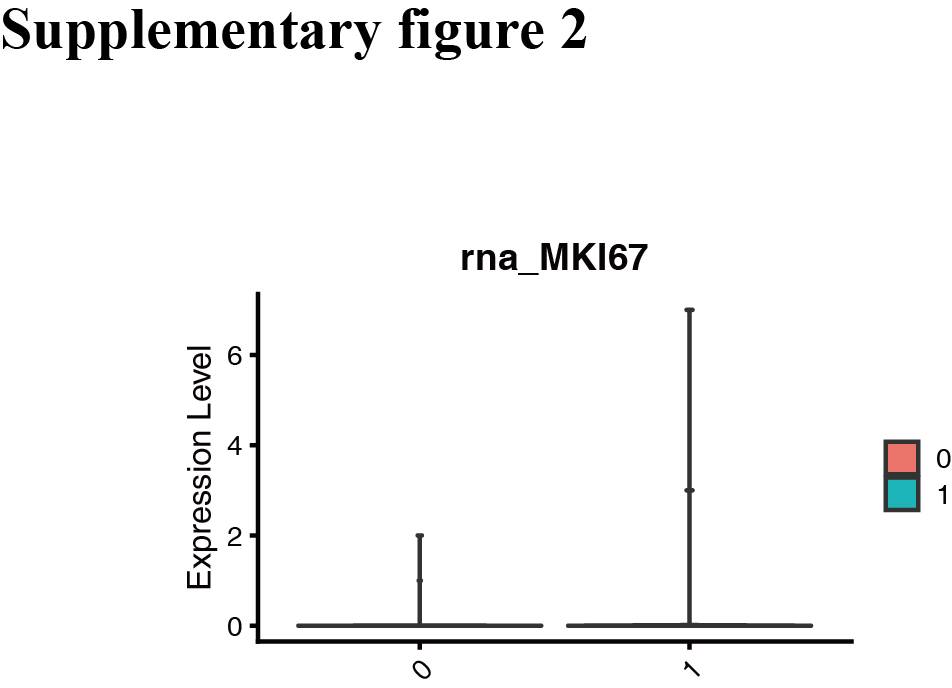

Supplement: Supplementary Figure 2 — Confirmative Analysis Of Plasma Cells. A violin plot showing the expression levels of MKI67 between plasma cell subpopulations. [file Image_2.jpg]

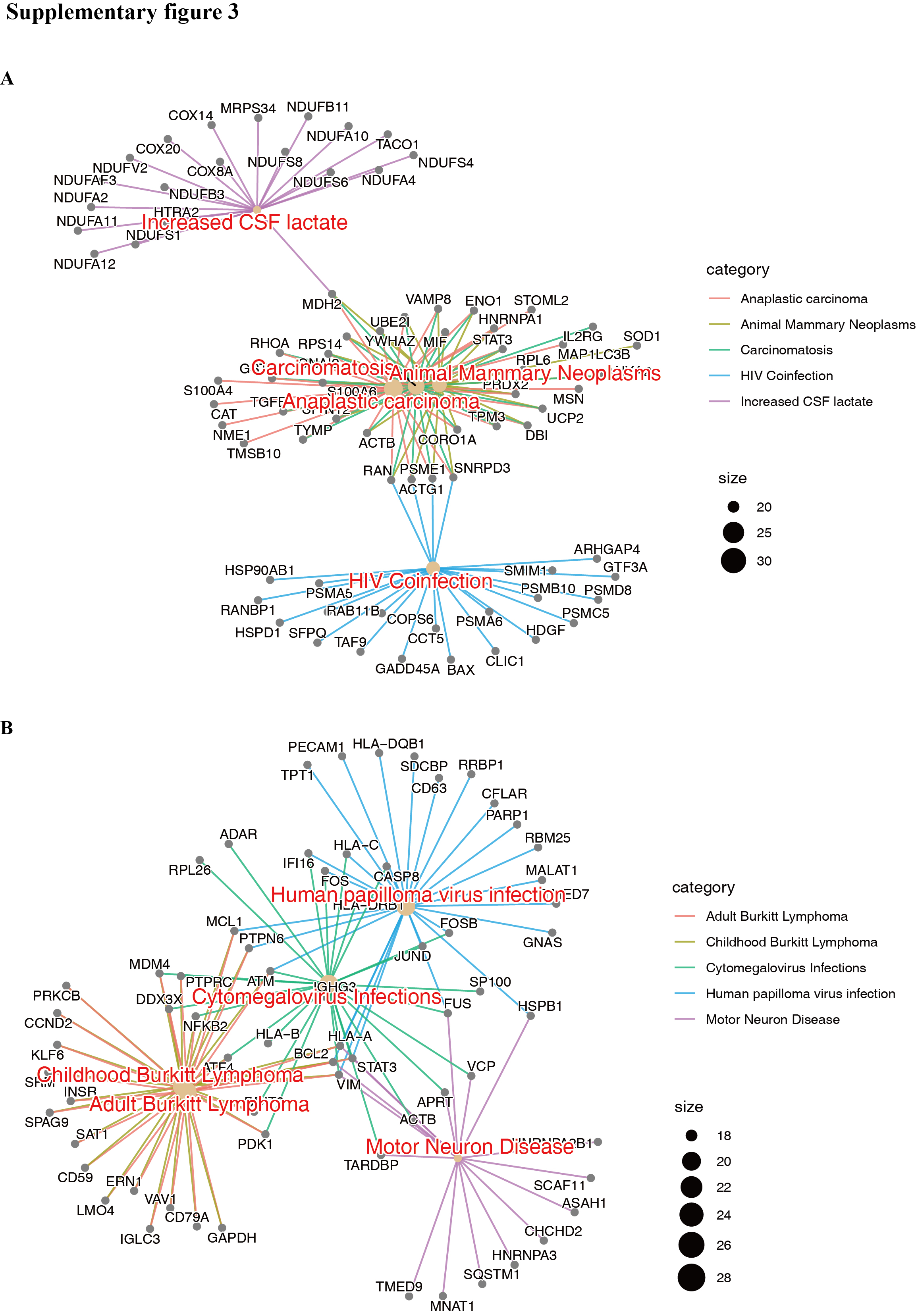

Supplement: Supplementary Figure 3 — Gene-Set-Enrichment Analysis of Plasma Cell Subpopulations. (A) Analysis of gene-concept-network in plasma cell subpopulation: plasma cell (RPS12). (B) Analysis of gene-concept-network in plasma cell subpopulation: plasma cell (IGHG4). [file Image_3.jpg]
